# TNF‐α induces up‐regulation of MicroRNA‐27a via the P38 signalling pathway, which inhibits intervertebral disc degeneration by targeting FSTL1

**DOI:** 10.1111/jcmm.16745

**Published:** 2021-06-30

**Authors:** Jie Shi, Shaoyi Wang, Qiting He, Kaiwen Liu, Wei Zhao, Qing Xie, Lei Cheng

**Affiliations:** ^1^ Department of Orthopaedic Qilu Hospital Cheeloo College of Medicine of Shandong University Jinan China; ^2^ Cheeloo College of Medicine Shandong University Jinan China; ^3^ NHC key Laboratory of Otorhinolaryngology Qilu Hospital Cheeloo College of Medicine Shandong University Jinan China; ^4^ Department of Pharmacy Qilu Hospital Cheeloo College of Medicine of Shandong University Jinan China

**Keywords:** chronic disease, degeneration, FSTL1, intervertebral disc, miRNA‐27a

## Abstract

The mechanism of intervertebral disc degeneration is still unclear, and there are no effective therapeutic strategies for treating this condition. miRNAs are naturally occurring macromolecules in the human body and have many biological functions. Therefore, we hope to elucidate whether miRNAs are associated with intervertebral disc degeneration and the underlying mechanisms involved. In our study, differentially expressed miRNAs were predicted by the GEO database and then confirmed by qPCR and in situ hybridization. Apoptosis of nucleus pulposus cells was detected by flow cytometry and Bcl2, Bax and caspase 3. Deposition of extracellular matrix was assessed by Alcian blue staining, and the expression of COX2 and MMP13 was detected by immunofluorescence, Western blot and qPCR. Moreover, qPCR was used to detect the expression of miR27a and its precursors. The results showed that miR27a was rarely expressed in healthy intervertebral discs but showed increased expression in degenerated intervertebral discs. Ectopic miR27a expression inhibited apoptosis, suppressed the inflammatory response and attenuated the catabolism of the extracellular matrix by targeting FSTL1. Furthermore, it seems that the expression of miR27a was up‐regulated by TNF‐α via the P38 signalling pathway. So we conclude that TNF‐α and FSTL1 engage in a positive feedback loop to promote intervertebral disc degeneration. At the same time, miR27a is up‐regulated by TNF‐α via the P38 signalling pathway, which ameliorates inflammation, apoptosis and matrix degradation by targeting FSTL1. Thus, this negative feedback mechanism might contribute to the maintenance of a low degeneration load and would be beneficial to maintain a persistent chronic disc degeneration.

## 1 BACKGROUND

Low back pain, a highly common physiological ailment that results in a large amount of medical expenses and reduced quality of life,^1^ is most often caused by intervertebral disc (IVD) degeneration (IDD).^2^ IDD involves a reduction in the number of nucleus pulposus (NP) cells, an imbalance of anabolism and catabolism in the extracellular matrix, and the inflammatory response.^3^, ^4^, ^5^ These various factors influence and promote each other, leading to an amplified cascade of IDD.^6^ However, these factors in the IVD must be tightly regulated so that IDD is not overactivated.^7^ In fact, IDD is a chronic pathological process. Most of the current research has focused on the positive feedback‐mediated enhancement of disc degeneration, but little is known about the mechanism of chronic degeneration. Therefore, we hope to explore the potential mechanism of IDD and to find new potential therapeutic targets for IDD.

MicroRNAs (miRNAs) are endogenous RNAs ~20 nt in length that act in a variety of biological capacities by binding to characteristic sequences in the 3′‐UTR of mRNAs to either degrade specific mRNAs or inhibit their translation into proteins.^8^ Because an miRNA can regulate multiple target genes and a target gene can be co‐regulated by multiple miRNAs, the biological effects of miRNAs are complex.^9^, ^10^ A growing number of studies have shown that miRNAs are involved in many physiological and pathological processes, such as cell proliferation and apoptosis,^11^, ^12^, ^13^ the regulation of tumour growth and development,^14^, ^15^, ^16^ the repair of damaged skin,^17^ normal body development^18^, ^19^ and the regulation of inflammation.^20^, ^21^, ^22^ More importantly, many miRNAs have been reported to inhibit the progression of pathological processes. Studies have shown that vesicular stomatitis virus (VSV) infection induces miR‐146a expression, which can suppress the production of type I IFN by targeting IL‐1R‐associated kinases 1 and 2 and TNFR‐associated factor 6.^23^ IFN‐induced miR27a expression results in negative feedback to reduce IFN production, which can restrict IFN‐induced inflammation.^24^ miR27a has been shown to protect against rheumatoid arthritis by inhibiting extracellular matrix breakdown.^25^ All of these studies indicated that the chronic process of disc degeneration may be closely regulated by miRNAs, but the underlying mechanism remains unclear.

Follistatin‐like protein 1 (FSTL1) was first identified by Shibanuma et al^26^ as a secreted glycoprotein that is involved in the physiological processes of growth and development^27^, ^28^ as well as in pathological processes such as pulmonary fibrosis,^29^ tumour development^30^, ^31^ and inflammation.^32^ Recent studies have shown that FSTL1 can promote the synthesis of matrix metalloproteinases related to the catabolism of the extracellular matrix,^33^ and other research has shown that FSTL1 is closely related to apoptosis.^34^ Our previous work revealed that FSTL1 is involved in disc degeneration and can promote inflammation in NP cells. We hypothesized that blocking the effects of FSTL1 specifically would reduce chronic inflammation of IVDs and thus delay IDD.

Here, we observed increased miR27a levels in the NP in patients with chronic disc degeneration. We also found that TNF‐α–mediated apoptosis, matrix degradation and inflammatory responses in NP cells were accompanied by an increase in miR27a expression through the p38 signalling pathway. When overexpressed, miR27a inhibited apoptosis, matrix degradation and inflammatory responses in NP cells by targeting FSTL1, leading to chronic degeneration. Therefore, we hypothesized that TNF‐α–induced increases in miR27a expression form a negative feedback loop to regulate disc degeneration by targeting FSTL1.

## 2 MATERIALS AND METHODS

### 2.1 Ethics statement

All patients with IDD between July 2019 and November 2019 at Qilu Hospital of Shandong University were enrolled in this study. Collection of human lumbar disc tissue samples adhered to medical ethics regulations and was approved by the Medical Ethical Committee of Qilu Hospital of Shandong University. This study was approved by the Qilu Hospital of Shandong University Review Board, and a written informed consent document was requested and received from all study participants.

### 2.2 LDH patients and controls

Degenerated human lumbar IVD samples were collected as surgical waste from patients undergoing spinal surgery in our hospital. Control (ie non‐degenerated) samples were obtained from patients suffering from lumbar fracture or scoliosis. Samples were collected upon approval of the local ethics committee. Before surgery, magnetic resonance imaging (MRI) was performed, and the extent of IDD was graded according to the classification system described by Pfirrmann et al^35^ The samples were divided into two groups: non‐degenerated (grade I/II), which comprised ten samples, and severely degenerated (grade IV/V), which had eleven samples. The characteristics of the patients are listed in Table 1.

**TABLE 1:** Patient information table

Group	Gender	Age	Pfirrmann grading
Control	M/F = 3:7	Average=59.9	GradeⅠ/Ⅱ
IDD	M/F = 4:7	Average=50.2	GradeⅣ/Ⅴ

### 2.3 Isolation and culture of human nucleus pulposus cells

Nucleus pulposus cells were extracted from the NP of twenty patients who underwent intervertebral discectomy. Disc samples from patients were digested with trypsin (Gibco, Thermo Fisher Scientific Inc) and collagenase II (Sigma‐Aldrich). Dulbecco's modified Eagle medium: nutrient mixture F‐12 (DMEM/F‐12; Gibco) culture medium supplemented with 10% foetal bovine serum (FBS; Gibco) and 1% penicillin/streptomycin were used to culture cells under standard incubation conditions (37°C, 5% CO2, 95% air). The cell culture medium was changed every 3 days, and the cells were passaged when they reached 90% confluence. Primary NP cells from passages 2, 3 or 4 were harvested and cultured in 6‐well, 12‐well or 24‐well plates at densities of 15 × 10^4^ cells/well, 10 × 10^4^ cells/well and 5 × 10^4^ cells/well, respectively, 1 day prior to treatment. miRNA control mimic or miR27a mimic (GenePharma) was transfected at a final concentration of 50 nmol/L with Lipofectamine 2000 (Invitrogen).

### 2.4 Data source and data analysis

The gene expression data sets analysed in this study were obtained from the GEO database (https://www.ncbi.nlm.nih.gov/geo/). After careful review, the gene expression profiles (GSE19943) were selected based on the GPL9946 platform (Exiqon human miRCURY LNA™ microRNA Array V11.0). All of the data were freely available online. R software and the limma package in R software were used to detect the differentially expressed genes (DEGs) between the degenerated and healthy samples, and the adjusted *P*‐value and |logFC| were calculated. Genes that met the cut‐off criteria—adjusted *P* < .05 and |logFC|<1.0—were considered DEGs. Statistical analysis was carried out for each data set.

### 2.5 In situ hybridization

Formalin‐fixed paraffin‐embedded (FFPE) tissue microarray sections were used for in situ hybridization (ISH) with double digoxigenin (DIG)‐labelled LNA probes labelled at both the 3′ and 5′ ends. LNA ISH on paraffin‐embedded tissue sections with probes specific for human miR27a was performed according to the manufacturer's instructions (Exiqon). ISH scoring was conducted by two independent observers (QTH and SYW) using conventional bright field microscopy, and differences in interpretation were reviewed for consensus.

### 2.6 Cell immunofluorescence

The NP cells were seeded into 24‐well plates at 5 × 10^4^ cells/well. After culture and treatment, the cells were fixed and incubated with primary antibodies overnight at 4°C and then with secondary antibodies for 1 hour. The nuclei were stained with DAPI before the samples were observed with a High Content Screening System (Opera Phenix, PerkinElmer).

### 2.7 Western blot analysis

Nucleus pulposus cell lysates were prepared in RIPA buffer (Beyotime Biotechnology). After the total protein concentrations were detected with a bicinchoninic acid (BCA) protein assay (Beyotime), an equal amount of protein from each sample was resolved by SDS‐PAGE on 10% SDS‐polyacrylamide gels and transferred to a polyvinylidene difluoride (PVDF) membrane for immunoblot analyses. The following primary antibodies were incubated with the membranes overnight at 4°C: rabbit anti‐FSTL1 (1:1000; Abcam, ab223287), rabbit anti‐MMP‐13 (1:3000; Abcam, ab51072), rabbit anti‐COX‐2 (1:2000; Abcam, ab15191), rabbit anti‐Bax (1:1000; affinity Biosciences, AF0120), rabbit anti‐caspase 3 (1:1000; affinity Biosciences, DF6879), rabbit anti‐Bcl2 (1:500, ProteinTech, 12789‐1‐AP), mouse anti‐β‐actin (1:10000; CST, 8H10D10) and mouse anti‐p38 (1:1000; Abcam, 31828). Unbound antibodies on the PVDF membranes were washed, secondary antibody (1:10000) was added, and the bands were visualized using an infrared laser imaging system (Li‐COR). ImageJ software (National Institutes of Health) was used for densitometry analysis.

### 2.8 Real‐time PCR

Total RNA was isolated from cultured NP cells and resected NP tissue using an RNeasy Kit (Qiagen). Reverse transcription was performed using a real‐time RT‐PCR Kit (Toyobo) according to the manufacturer's instructions. Real‐time PCR was performed with SYBR Green I dye, which is used to monitor DNA synthesis. The reactions were carried out on a PCR instrument (ABI‐7900). Target gene expression was normalized to that of β‐actin. For each target gene, the experiment was repeated three times. Sequence‐specific primers are listed in Table 2.

**TABLE 2:** Primer sequences for real‐time PCR

Primer	F	R
miR27A	AGGCAGGTTCACAGTGGCTAAG	GTGCAGGGTCCGAGGT
U6	CAGCACATATACTAAAATTGGAACG	ACGAATTTGCGTGTCATCC
Pri‐miR27	ATTTCCAACCGACCCTGAGC	CAGGATGGCAGGCAGACAG
Pre‐miR27	CTGAGGAGCAGGGCTTAGCTGCTTG	GCGGAACTTAGCCACTGTGAACACG
Β‐Actin	CATGTACGTTGCTATCCAGGC	CTCCTTAATGTCACGCACGAT
MMP13	ATTAAGGAGCATGGCGACTTCT	GCCCAGGAGGAAAAGCATGA
COX2	TCCCTTGGGTGTCAAAGGTAAA	TGGCCCTCGCTTATGATCTG

For miRNA quantification, the GoScript Reverse Transcription System Kit (Promega) was used with the stem‐loop primer. For each sample, the relative miRNA level was normalized to that of U6. The forward and reverse primers for miR27a and U6 are listed in Table 2. Relative expression levels of miRNA were detected using an ABI‐7900.

### 2.9 Alcian blue staining

Alcian blue staining was applied to detect glycosaminoglycan (GAG) sulphate deposition. NP cells were washed twice with PBS and subsequently fixed with 4% paraformaldehyde in PBS for 10 minutes at room temperature. Fixed cells were washed 6 times with PBS and air‐dried for storage until they were incubated with 1% Alcian blue (Sigma) in 0.1 M HCl at room temperature overnight. Cells were washed 6 times with PBS to remove excess Alcian blue and allowed to air‐dry in the dark overnight.

### 2.10 Flow cytometry analysis

The cells in the 6‐well plate were washed three times with cooled PBS and then collected in a centrifuge tube for further operation. Flow cytometry is performed according to manufacturer's instructions (E‐CK‐A211). The cell was resuspended in 1× binding buffer at a concentration of 1 × 106 cells/mL. Next, 100 µL of this solution was transferred into a 5‐mL culture tube, and 5 µL FITC Annexin Ⅴ (or PE Annexin Ⅴ) and 5 µL PI were added. The cells were gently vortexed and incubated for 15 minutes at room temperature in the dark. Finally, 400 µL of 1× binding buffer was added to the tube. Flow cytometry was then conducted within 1 hour with a BD Accuri C6 Plus Flow Cytometer. Data were analysed by FlowJo V10 software (BD).

### 2.11 Statistical analysis

All data are expressed as the means ± SD of results derived from three independent experiments performed in triplicate. Statistical analysis was performed by Student's *t* test. A difference was considered significant if *P* < .05.

## 3 RESULTS

### 3.1 The expression of miR27a is elevated in degenerated IVDs

**FIGURE 1:**
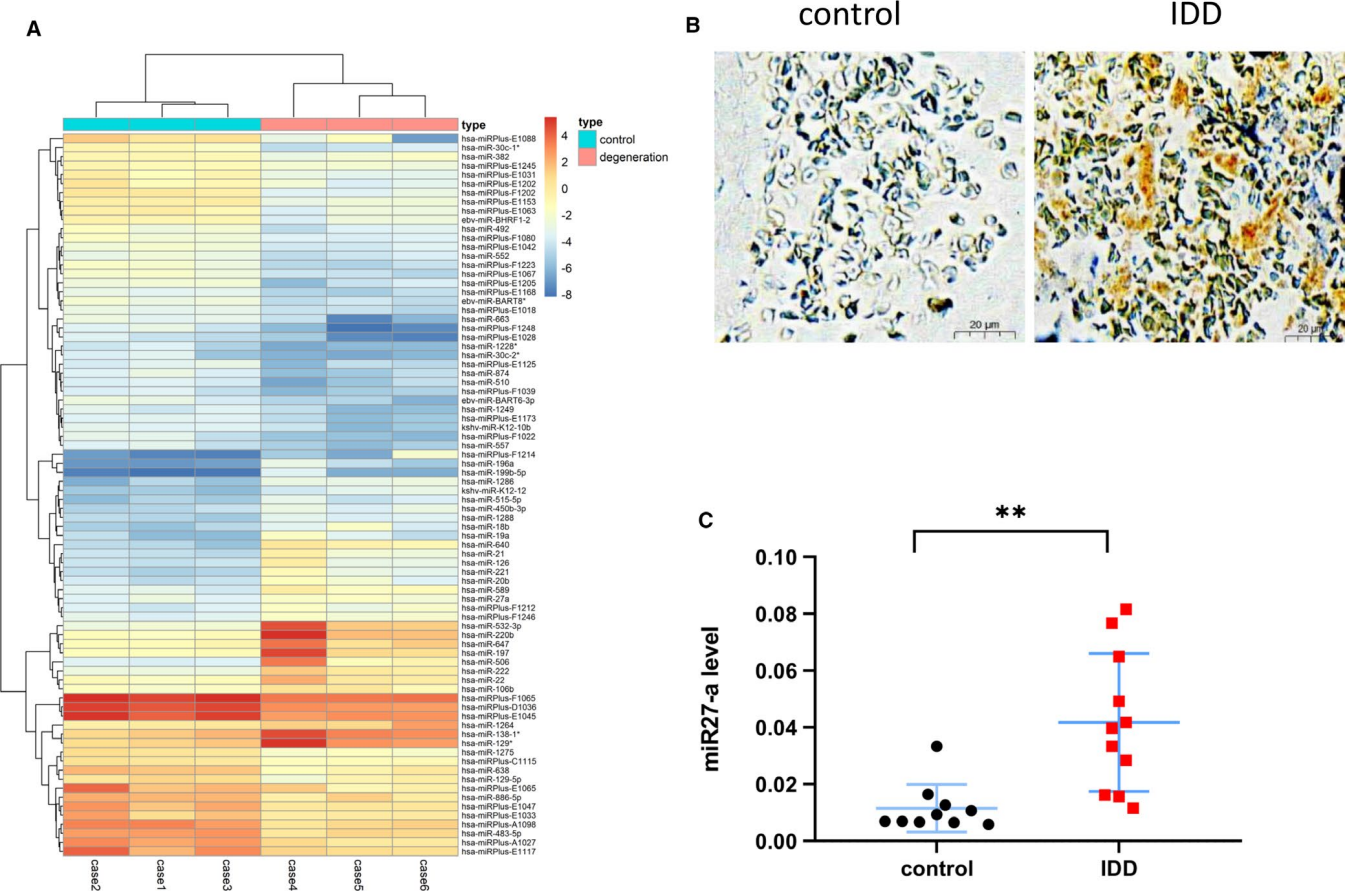
The expression of miR27a is increased in degenerating IVDs. (A) Many miRNAs are differentially expressed based on analysis of the GEO data set. (B) The expression of miR27a in 3 control and 3 IDD patients was detected by ISH. (C) The expression of miR27a in 10 control and 11 IDD patients was detected by qPCR *P* < .05 was considered to be statistically significant. Representative images are shown

To determine the miRNA(s) involved in IDD, we analysed the GSE19943 chip data from the GEO database. Compared with the control group, the IDD group showed differential expression of multiple miRNAs (Figure 1A), with a total of 30 miRNAs with increased expression and 50 miRNAs with decreased expression. Because up‐regulated genes are more likely to play an active role in IDD, we further analysed the up‐regulated miRNAs. It is interesting to note that there is a wealth of evidence that miR27a plays an important protective role in other diseases; thus, we decided to explore whether miR27a plays a protective role in IDD. First, we performed ISH (in situ hybridization) to detect miR27a expression in NP tissue from 3 patients with IDD and 3 individuals with normal IVDs. The results indicated that miR27a is natively expressed in human NP tissue (mainly in the cytoplasm) and that miR27a expression is higher in degenerated IVDs than in normal IVD (Figure 1B). Moreover, we isolated total RNA in NP tissues from 11 patients with IDD and 10 patients with normal IVDs and then used qPCR to detect the difference in miR27a expression. The results revealed that, in NP tissues, miR27a expression in patients with IDD was significantly higher than that in patients with normal IVDs (Figure 1C). These results suggest that miR27a is involved in the process of IDD and that its expression is elevated in degenerated IVDs.

### 3.2 Overexpression of miR27a inhibits apoptosis and the production of inflammatory cytokines in nucleus pulposus cells

**FIGURE 2:**
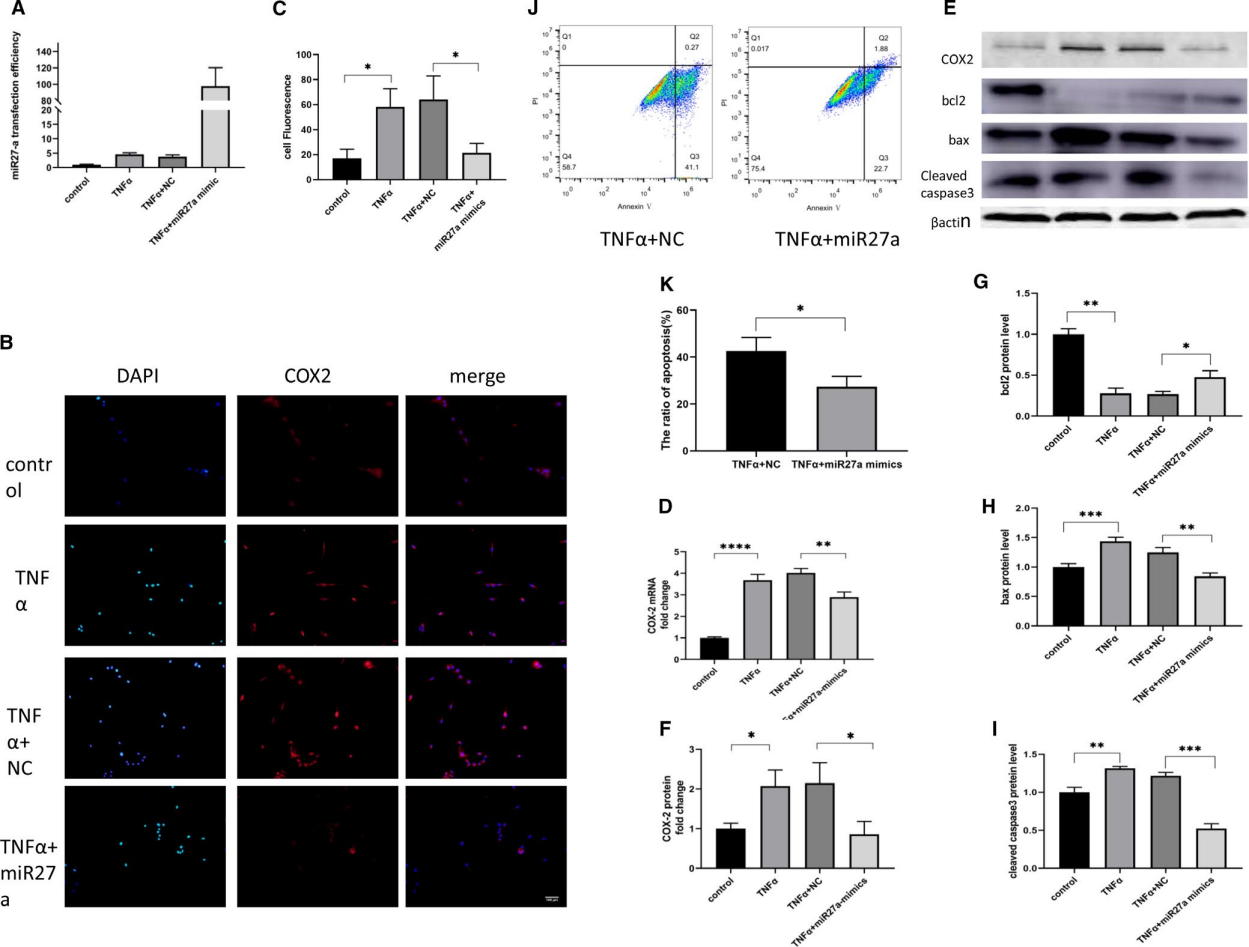
Overexpression of miR27a in nucleus pulposus cells inhibits apoptosis and the production of inflammatory cytokines. (A) The transfection efficiency of miR27a as detected by qPCR. (B, C, D, E and F) NP cells were transfected with miR27a mimics or NC for 24 h and then treated with 10 ng/mL TNF‐α. After 24 h, COX2 expression was detected by immunofluorescence (B, C), qPCR (D) and Western blotting (E, F). (E, G, H, I) NP cells were transfected with miR27a mimics or NC for 24 h and then treated with 10 ng/mL TNF‐α. After 24 h, Bcl2, Bax and caspase 3 expression was detected by Western blotting. (J, K) NP cells were transfected with miR27a mimics or NC for 24 h and then treated with 10 ng/mL TNF‐α. After 24 h, apoptosis was detected by flow cytometry. Statistical analysis was based on at least three different biological samples and three technical replicates; *P* < .05 was considered to be statistically significant. Representative images are shown

To investigate whether miR27a affects the apoptosis and inflammation of NP cells, we treated NP cells with TNF‐α to create a model of IDD. One day prior to TNF‐α treatment, NP cells were either transfected with miR27a mimics or NC (negative control) mimic or left untreated. qPCR was used to evaluate the transfection efficiency of miR27a, and the results showed that miR27a expression in miR27a mimic group was significantly increased (increased to 98‐fold compared with control) (Figure 2A). To investigate the inflammatory changes in NP cells after TNF‐α treatment, cellular immunofluorescence was used to detect COX2 expression. TNF‐α stimulation increased COX2 protein synthesis in NP cells, and a high level of miR27a expression reduced COX2 synthesis (reduced to 0.34‐fold compared with negative control) (Figure 2B,C). This suggests that miR27a in NP cells can mitigate its own inflammatory response. For further confirmation, we used Western blotting and qPCR to quantify COX2 synthesis and observed the same trend (reduced to 0.4‐ and 0.7‐fold compared with negative control, respectively) (Figure 2D,E,F). To investigate whether miR27a protects NP cells from apoptosis, flow cytometry was used to assess apoptosis rates (the apoptosis rate decreased to 0.6‐fold compared with the negative control group)(Figure 2J,K), and Western blotting was used to measure the levels of Bax, Bcl2 and caspase 3 proteins to assess cell apoptosis (the expression in the miR27a mimic group was 0.7‐, 1.8‐, 0.4‐fold compared with negative control group, respectively) (Figure 2E,G,H,I). The data indicated that miR27a could reduce TNF‐α–induced apoptosis of NP cells, suggesting that miR27a plays an antiapoptotic role in degenerated IVDs.

### 3.3 Overexpression of miR27a inhibits the production of matrix metalloproteinases and increases extracellular matrix deposition

**FIGURE 3:**
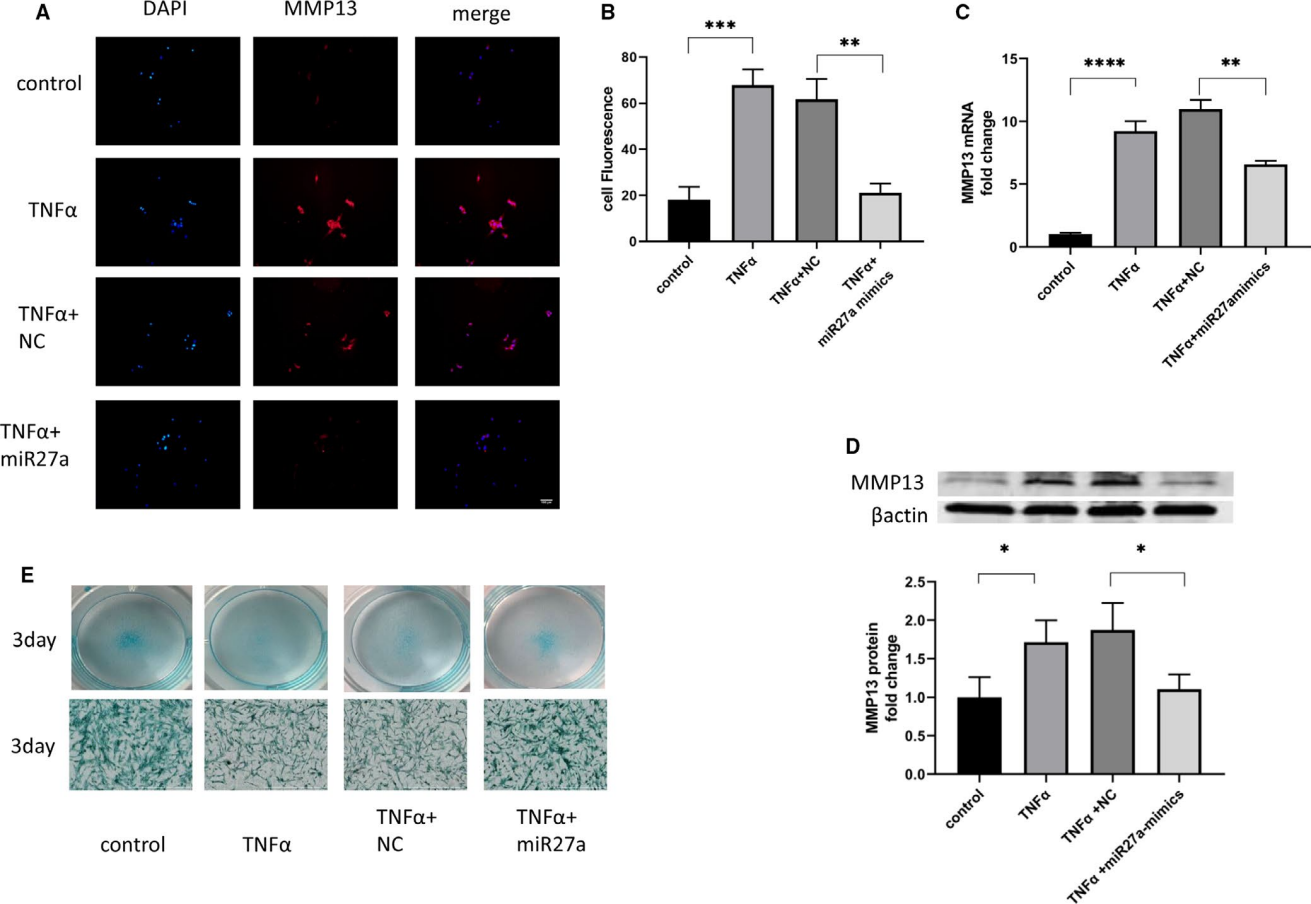
Overexpression of miR27a inhibits the production of matrix metalloproteinases and increases the deposition of extracellular matrix. (A, B, C, D) NP cells were transfected with miR27a mimics or NC for 24 h and then treated with 10 ng/mL TNF‐α. After 24 h, MMP13 expression was detected by immunofluorescence (A), qPCR (B) and Western blotting (C, D). (E) NP cells were transfected with miR27a mimics or NC for 24 h and then treated with 10 ng/mL TNF‐α. After 3 d, extracellular matrix deposition was detected by Alcian blue staining. Statistical analysis was based on at least three different biological samples and three technical replicates; *P* < .05 was considered to be statistically significant. Representative images are shown

To explore whether miR27a plays a protective role in the catabolism of the extracellular matrix, cellular immunofluorescence was used to detect MMP13 expression in NP cells. TNF‐α stimulation increased MMP13 protein synthesis in NP cells, and a high level of miR27a expression abrogated this increase (reduced to 0.33‐fold) (Figure 3A,B). This suggests that miR27a expression in NP cells slows or inhibits degradation of the extracellular matrix. To confirm this notion, we used Western blotting and qPCR to quantify the amount of MMP13 synthesized by NP cells, and the results showed the same trend (reduced to 0.6, 0.6‐fold, respectively) (Figure 3C,D). We then semiquantified the amount of extracellular matrix using Alcian blue staining. After 4 days of TNF‐α stimulation, more GAG deposition was observed in the miR27a‐overexpressing group than in the miR27a‐underexpressing group (Figure 3E). These results suggest that miR27a in NP cells can protect against the catabolism induced by TNF‐α.

### 3.4 miR27a inhibits apoptosis, the inflammatory response and matrix degradation by targeting FSTL1

**FIGURE 4:**
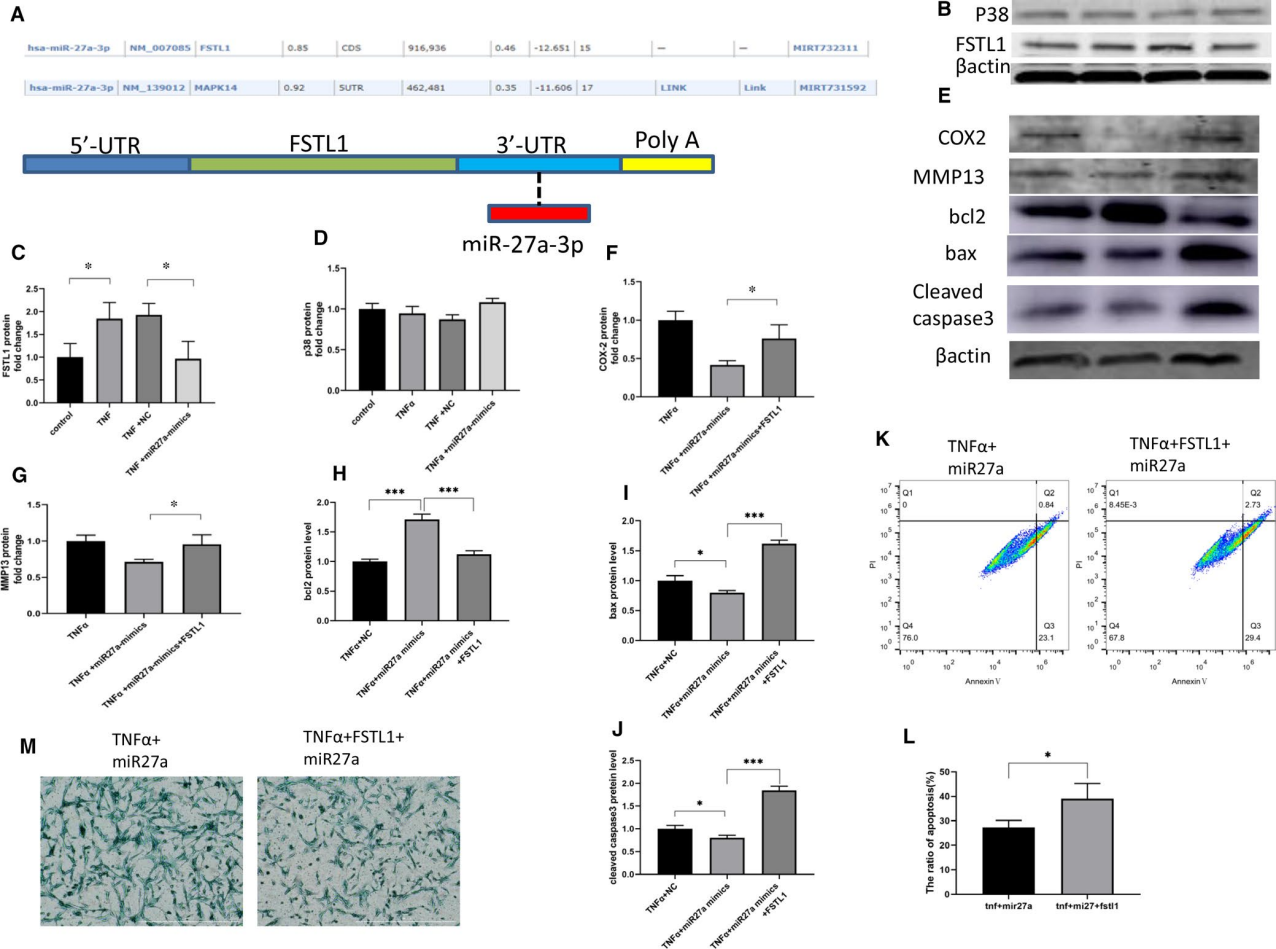
MiR27a inhibits apoptosis, the inflammatory response and matrix degradation by targeting FSTL1. (A) The target genes of miR27a were predicted by miRWalk. (B, C, D) NP cells were transfected with miR27a mimics or NC for 24 h and then treated with 10 ng/mL TNF‐α. After 24 h, FSTL1 (B, C) and P38 (B, D) protein expression was detected by Western blotting. (E, F, G, H, I, J) NP cells were transfected with miR27a mimics for 24 h and then treated with 10 ng/mL TNF‐α alone or in combination with 100 ng/mL rhFSTL1. After 24 h, MMP13 (E, G), Bcl2 (E, H), Bax (E, I), caspase 3 (E, J) and COX2 (E, F) protein expression was detected by Western blotting. (K, L) NP cells were transfected with miR27a mimics for 24 h and then treated with 10 ng/mL TNF‐α alone or in combination with 100 ng/mL rhFSTL1. After 24 h, apoptosis was detected by flow cytometry. (M) NP cells were transfected with miR27a mimics for 24 h and then treated with 10 ng/mL TNF‐α alone or in combination with 100 ng/mL rhFSTL1. After 3 d, extracellular matrix deposition was detected by Alcian blue staining. Statistical analysis was based on at least three different biological samples and three technical replicates; *P* < .05 was considered to be statistically significant. Representative images are shown

Previous studies have shown that FSTL1 can promote IDD, and activation of the P38 pathway has also been reported to promote disc degeneration. In addition, other researchers reported that although P38 and FSTL1 are the target genes of miR27a.^25^, ^36^ miR27a inhibits target gene translation by binding to the UTR region of its target mRNA, and then, the target genes of miR27a were predicted to be P38 and FSTL1 by miRWalk (Figure 4A). Overexpression of miR27a can down‐regulate the expression of the target gene, thereby blocking downstream signalling. Therefore, we investigated whether the mechanism by which miR27a plays an antidegenerative role functions by targeting FSTL1 or P38. Western blotting was used to detect the expression of FSTL1 and P38 to investigate whether miR27a down‐regulated either FSTL1 or P38 expression in NP cells. The data showed that miR27a down‐regulated the expression of FSTL1 in NP cells (reduced to 0.5‐fold compared with negative control group) (Figure 4B,C) but had no effect on P38 expression in NP cells (Figure 4B,D). Then, a functional recovery experiment was performed to confirm that miR27a plays an antidegenerative role through FSTL1, and the results showed that after rhFSTL1 stimulation, the protective effect of miR27a was eliminated, the expression of related genes be restored (Figure 4E,F,G,H,I,J), GAG deposition decreased and apoptosis increased (Figure 4K,L,M). These results suggest that miR27a inhibits apoptosis, the inflammatory response and matrix degradation by targeting FSTL1.

### 3.5 TNF‐α up‐regulates miR27a expression through the P38 pathway

**FIGURE 5:**
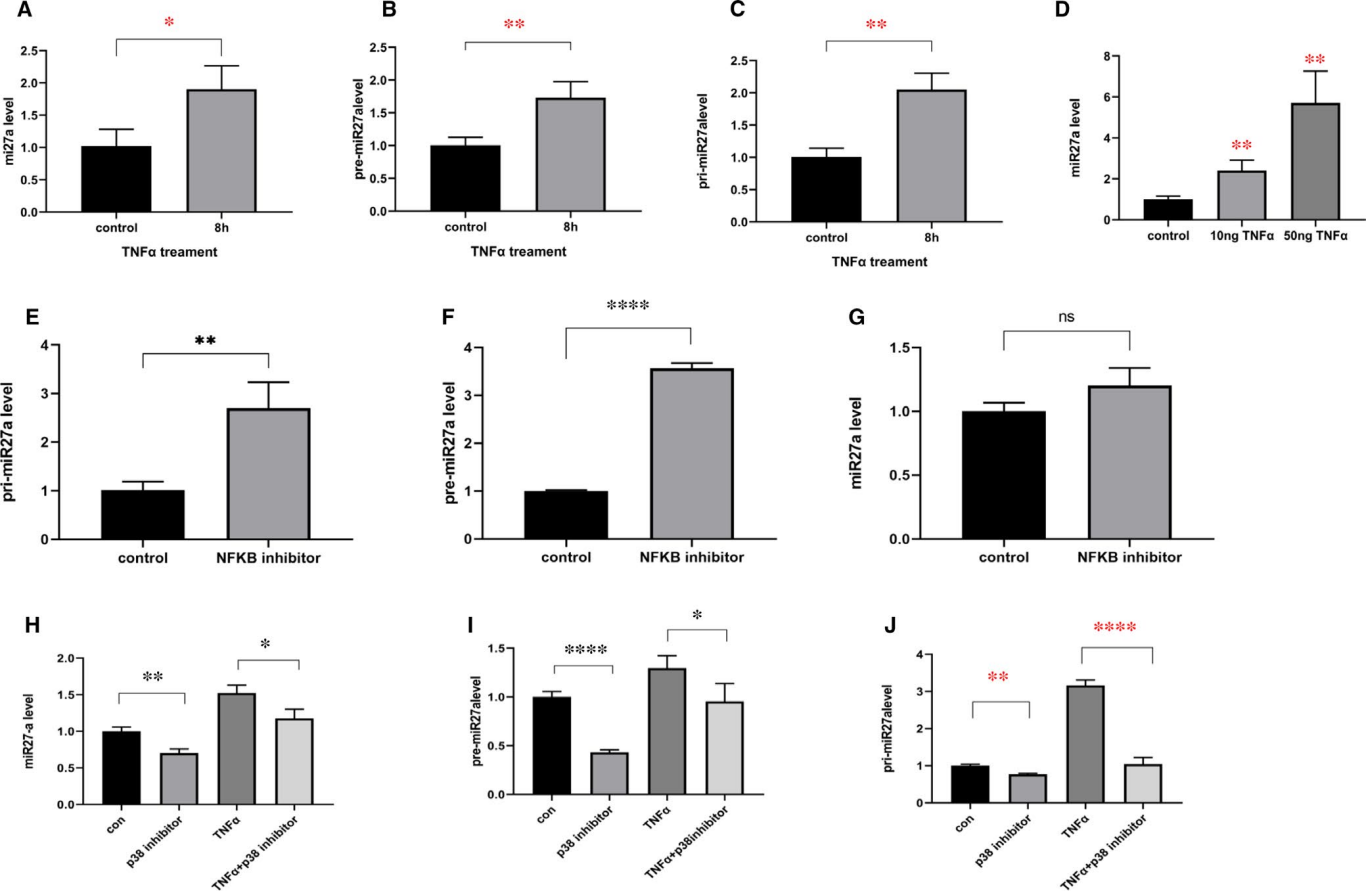
TNF‐α up‐regulates miR27a expression through the P38 pathway. (A, B, C) NP cells were treated with TNF‐α for 8 h, and miR27a (A), pre‐miR27a (B) and pri‐miR27a (C) were measured by qPCR. (D) NP cells were treated with different concentrations of TNF‐α, and miR27a expression was measured by qPCR after 8 h. (E, F, G) NP cells were treated with an inhibitor of NF‐κB (5 µmol/L) for 8 h, and pri‐miR27a (E), pre‐miR27a (F) and miR27a (G) expression was measured by qPCR. (H&I&J) NP cells were treated with an inhibitor of P38 at 10 µmol/L for an hour followed by treatment with TNF‐α for 8 h. Then, miR27a (H), pre‐miR27a (I) and pri‐miR27a (J) expression was measured by qPCR. Statistical analysis was based on at least three different biological samples and three technical replicates; *P* < .05 was considered to be statistically significant. Representative images are shown

Next, we sought to determine why miR27a expression is increased in degenerated IVDs by conducting in vitro experiments to explore the mechanism by which miR27a expression is elevated. We used TNF‐α–treated NP cells to establish a model of IDD, and miR27a expression was increased in the TNF‐α–treated group at 8 hours compared with that in the control group at the same time (increased to 1.9‐fold compared with control) (Figure 5A). To explore whether miR27a is changed during transcription or during posttranscriptional transport, shear or degradation, we further examined the precursors and initial transcripts of miR27a. We found that at 8 hours, pri‐miR27a and pre‐miR27a presented the same trend in altered expression as that of miR27a (increased to 1.7, 2‐fold compared with control, respectively)(Figure 5B,C). Furthermore, we treated NP cells with different concentrations of TNF‐α and found that the expression level of miR27a was dose‐dependent. The higher the concentration of TNF‐α is, the higher the expression level of miR27a (compared with the control group, the expression of miR27a was increased to 2.4‐fold at 10ng and 5.7‐fold at 50ng TNF‐α) (Figure 5D), which indicates that more severe degeneration of NP cells corresponds to higher expression levels of miR27a. To explore the pathway regulating miR27a expression, inhibitors of NF‐κB or of P38 were used. The results showed that the expressions of pri‐miR27a and pre‐miR27a were increased (increased to 22, 3.5‐fold compared with control, respectively) (Figure 5E,F), but there was no significant difference in miR27a expression between the control group and the NF‐κB treatment group (Figure 5G), suggesting that the NF‐κB signalling pathway does not significantly influence miR27a expression. However, compared with the control group, the P38 inhibitor treatment group had decreased expression levels of miR27a, pri‐miR27a and pre‐miR27a (reduced to 0.7‐, 0.7‐, 0.4‐fold, respectively) (Figure 5H,I,J), and the same trend was observed upon TNF‐α stimulation (reduced to 0.7‐, 0.3‐, 0.7‐fold, respectively) (Figure 5H,I,J). This indicates that TNF‐α can up‐regulate miR27a expression through the P38 pathway.

## 4 DISCUSSION

**FIGURE 6:**
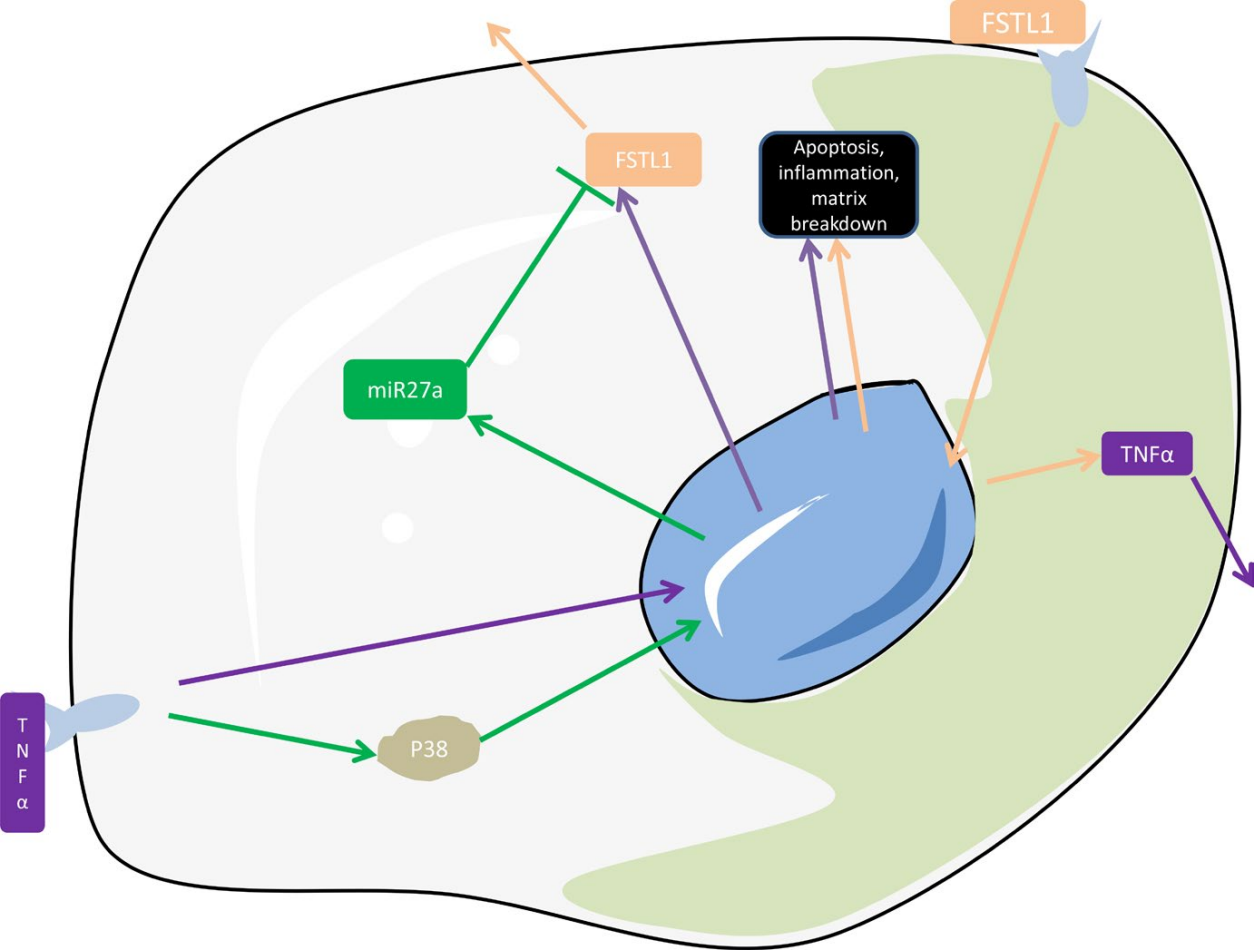
Both FSTL1 and TNF‐α can mediate IDD model in vitro. FSTL1 and TNF‐α promote each other and form a positive feedback loop to further progress IDD. While TNF‐α causes apoptosis, an inflammatory response and matrix degradation, it also up‐regulates miR27a expression, which can inhibit all three activities by targeting FSTL1

Intervertebral disc degeneration develops via a series of changes mainly involving apoptosis, the inflammatory response and extracellular matrix degradation. In the early stage of IDD, the activities of and cytokines secreted from cells are altered. For example, an increase in MMP13 levels may lead to increased extracellular matrix degradation, decreased extracellular matrix synthesis and decreased NP water content. Previous studies have shown that disruptions in extracellular matrix metabolism are often accompanied by disorders of homeostatic cell function.^37^ The increase in inflammatory factors leads to an increase in extracellular matrix degradation,^38^, ^39^ forming a positive feedback loop of cell reduction, extracellular matrix degradation and aggravated inflammation, when then cascades into the degeneration of the NP within the IVD (Figure 6). However, chronic disc degeneration is a tightly regulated process in which the cascade of extracellular matrix decomposition, apoptosis and inflammation is tightly regulated so as not to cause excessive acute injury to the body. Unlike previous studies that focused on the mechanisms that promote degeneration, our study reveals the underlying mechanisms of the body's own resistance to degeneration, miR27a acted as a regulator and was up‐regulated by TNF‐α to restrain excessive degeneration.

In our study, we offer a possible explanation by revealing the association between miR27a and IDD and came to the following conclusions: (a) miR27a is expressed in human IVD tissues in NP cells, with very low expression in individuals with non‐degenerated IVDs but higher expression in patients with IDD; (b) miR27a down‐regulates TNF‐α–induced expression of inflammatory genes and reduces TNF‐α–induced apoptosis of NP cells; (c) miR27a down‐regulates the TNF‐α–induced expression of genes related to the breakdown of extracellular matrix and increases the deposition of extracellular matrix; (d) miR27a inhibits NP cell apoptosis, matrix degradation and inflammation by targeting FSTL1; and (e) TNF‐α–induced IDD promotes miR27a expression through the P38 pathway.

In previous reports, miRNAs have been shown to inhibit inflammatory responses^40^ or biometabolism^41^ through negative feedback. In this study, we hoped to elucidate the potential mechanism for tightly regulating the progression of disc degeneration from the perspective of miRNA activity. First, we used the GEO database and discovered 50 down‐regulated miRNAs and 30 up‐regulated miRNAs. Since up‐regulated miRNAs are more likely to play an active role in pathologies, we analysed the most up‐regulated miRNAs and, combined with data from previous reports, identified miR27a as a potential modulator of the inflammatory response and catabolism.^36^, ^42^ Then, we verified miR27a expression with ISH and qPCR and found that miR27a was expressed at low levels in non‐degenerated disc tissues but at increased levels in degenerated disc tissues, indicating that miR27a may play an important role in disc degeneration.

Previous studies have shown that disc degeneration involves apoptosis of NP cells, NP tissue inflammation and extracellular matrix degradation.^43^, ^44^, ^45^ As previously reported, when TNF‐α was used to stimulate NP cells, there were increases in apoptosis, synthesis of proteases involved in matrix degradation and synthesis of inflammatory genes; by contrast, miR27a can reverse these effects, indicating that miR27a can slow the progression of and protect against IDD. Subsequent experiments showed that miR27a protects IVDs by targeting FSTL1 to restrain the activation of the degeneration cascade. As shown in our previous studies, FSTL1 promotes matrix degradation and the synthesis of inflammatory factors such as TNF‐α,^46^ which in turn promotes the synthesis of FSTL1 through a positive feedback loop. Therefore, we speculated that miR27a inhibited further degeneration by blocking FSTL1‐driven amplification of the signalling cascade.

Upon exploring the potential mechanism by which increased miR27a expression affects patients with IDD, we found that TNF‐α stimulated NP cells, leading to elevated miR27a expression, and further experiments revealed that TNF‐α promoted the transcription of miR27a through the P38 pathway. Our results suggest that the regulation of miR27a by the p38 pathway may occur at the transcriptional level, but the direct regulatory factors have not been found yet, and we will further explore it in future studies. However, although NF‐κB has been reported to be involved in many biological processes in many literatures,^47^, ^48^ our results show that NFκB inhibitors only increase the expression of the precursor miR27a, but have no significant effect on miR27a, this may be due to the shear and degradation of the precursor miR27a. Interestingly, TNF‐α is an important factor in disc degeneration^49^, ^50^; thus, these results suggest that TNF‐α induces apoptosis, the inflammatory response and extracellular matrix degradation and promotes FSTL1 expression when IDD occurs. FSTL1 is activated by positive feedback to promote TNF‐α expression, the inflammatory response and the degeneration cascade, while TNF‐α up‐regulates miR27a to regulate degeneration through negative feedback (Figure 6). We hypothesized that because miR27a is expressed at a relatively low level in the human body, the disc would maintain low levels of degeneration.

In summary, our study reveals the mechanism of chronic disc degeneration from the perspective of miRNAs. MiR27a, which is up‐regulated by TNF‐α through the P38 pathway, blocks disc degeneration by targeting FSTL1 and can inhibit disc degeneration when expressed at high levels, thus providing a new potential therapeutic target for future treatment.

## CONFLICT OF INTEREST

The authors declare that they have no competing interests.

## AUTHORS CONTRIBUTION

**Jie Shi:** Conceptualization (equal); Formal analysis (equal); Project administration (equal); Writing‐original draft (equal). **Shaoyi Wang:** Data curation (equal); Methodology (equal); Visualization (equal); Writing‐original draft (equal). **Qiting He:** Formal analysis (equal); Methodology (equal); Software (equal). **Kaiwen Liu:** Methodology (equal); Writing‐original draft (equal); Writing‐review & editing (equal). **Wei Zhao:** Formal analysis (equal); Methodology (equal); Validation (equal). **Qing Xie:** Conceptualization (equal); Data curation (equal); Formal analysis (equal); Methodology (lead); Writing‐review & editing (lead). **Lei Cheng:** Conceptualization (lead); Data curation (lead); Funding acquisition (lead); Methodology (equal); Project administration (equal); Writing‐review & editing (equal).

## ETHICAL APPROVAL

This study was approved by medical ethics regulations of the Medical Ethical Committee of Qilu Hospital of Shandong University (Jinan, China), and informed consent was not required.

## Data Availability

The datasets used and/or analysed of this study are available from authors on reasonable request.
